# Disseminated Breast Cancer Cells Acquire a Highly Malignant and Aggressive Metastatic Phenotype during Metastatic Latency in the Bone

**DOI:** 10.1371/journal.pone.0047587

**Published:** 2012-11-15

**Authors:** Carolyn G. Marsden, Mary Jo Wright, Latonya Carrier, Krzysztof Moroz, Brian G. Rowan

**Affiliations:** 1 Department of Structural and Cellular Biology, The Louisiana Cancer Research Consortium, Tulane University Health Sciences Center, New Orleans, Louisiana, United States of America; 2 Department of Surgery, The Louisiana Cancer Research Consortium, Tulane University School of Medicine, New Orleans, Louisiana, United States of America; 3 Section of Surgical Pathology and Cytopathology, Louisiana Cancer Research Consortium, Tulane University School of Medicine, New Orleans, Louisiana, United States of America; University of Medicine and Dentistry of New Jersey, United States of America

## Abstract

**Background:**

Disseminated tumor cells (DTCs) in the bone marrow may exist in a dormant state for extended periods of time, maintaining the ability to proliferate upon activation, engraft at new sites, and form detectable metastases. However, understanding of the behavior and biology of dormant breast cancer cells in the bone marrow niche remains limited, as well as their potential involvement in tumor recurrence and metastasis. Therefore, the purpose of this study was to investigate the tumorigenicity and metastatic potential of dormant disseminated breast cancer cells (prior to activation) in the bone marrow.

**Methodology/Principal Findings:**

Total bone marrow, isolated from mice previously injected with tumorspheres into the mammary fat pad, was injected into the mammary fat pad of NUDE mice. As a negative control, bone marrow isolated from non-injected mice was injected into the mammary fat pad of NUDE mice. The resultant tumors were analyzed by immunohistochemistry for expression of epithelial and mesenchymal markers. Mouse lungs, livers, and kidneys were analyzed by H+E staining to detect metastases. The injection of bone marrow isolated from mice previously injected with tumorspheres into the mammary fat pad, resulted in large tumor formation in the mammary fat pad 2 months post-injection. However, the injection of bone marrow isolated from non-injected mice did not result in tumor formation in the mammary fat pad. The DTC-derived tumors exhibited accelerated development of metastatic lesions within the lung, liver and kidney. The resultant tumors and the majority of metastatic lesions within the lung and liver exhibited a mesenchymal-like phenotype.

**Conclusions/Significance:**

Dormant DTCs within the bone marrow are highly malignant upon injection into the mammary fat pad, with the accelerated development of metastatic lesions within the lung, liver and kidney. These results suggest the acquisition of a more aggressive phenotype of DTCs during metastatic latency within the bone marrow microenvironment.

## Introduction

Once considered the final step during cancer progression, recent evidence implicates metastasis as an early event in breast cancer [1]–[4]. Disseminated tumor cells (DTCs) may be present at distant sites at the time of primary diagnosis of breast cancer in patients that exhibit no outward signs of clinical metastases. As a preferential site of metastasis for breast cancer [5], the detection of DTCs in the bone of breast cancer patients has become an important prognostic tool. It is estimated that DTCs can be detected in the bone marrow for up to 40% of breast cancer patients using the current detection technology [6], [7]. As a strong independent prognosticator, patients with DTCs in the bone marrow have an overall worse prognosis, as well as a higher propensity for local and distant relapse, compared to patients without DTCs in the bone marrow [8]–[10]. Despite the clinical significance of DTCs in the bone marrow, the biological relevance remains controversial [11].

Although DTCs can be detected in the bone marrow of early breast cancer patients, clinical manifestation of bone metastasis and/or recurrence often does not emerge for years or even decades after initial diagnosis [4], [12]. The lag time between detection of DTCs and manifestation of disease indicates the cells have become dormant, persisting as viable but non-proliferating cells [4], [13], [14]. The mechanisms by which the cells enter a dormant state can be intrinsic, a result of genetic and/or epigenetic modifications, or as a consequence of the microenvironment in which the cells reside [15]. Studies have shown significantly less chromosomal aberrations in DTCs in the bone marrow as compared to cells in the primary tumor [1], [3], [16] suggesting DTCs have not acquired the necessary genetic alterations to overcome growth restraints. However, early DTCs have also been shown to be genomically very unstable [17], [18]. These conflicting reports concerning the intrinsic properties of DTCs indicates it is unlikely intrinsic mechanisms alone can account for the long dormancy periods observed by DTCs in the bone marrow. Alternatively, the bone marrow microenvironment has been implicated as a supportive niche for the existence of disseminated breast cancer cells in a dormant state [19], [20]. Breast cancer cells localized close to the endosteum, the interface of bone and marrow which serves as a supportive niche for hematopoietic stem cells and a frequent site of cancer cell dissemination, were shown to have long doubling times suggesting a possible quiescent state [21]. Intercellular communication through gap junctions between breast cancer cells and the bone marrow stroma close to the endosteum has recently been suggested to play a role in the maintenance of a dormant state [22]. Furthermore, *in vitro* studies have demonstrated an inhibitory effect on proliferation and acquisition of an invasive mesenchymal phenotype of breast cancer cells upon co-culture with bone marrow stroma isolated from breast cancer patients [15]. These findings illustrate the significance of the cellular interactions within the bone marrow microenvironment and the subsequent effects on the phenotype of disseminated breast cancer cells.

Recent reports have presented data supporting the bi-directional flow of DTCs, demonstrating targeted homing of DTCs to tumors present in the mammary fat pad and accelerated tumor progression upon colonization by the DTCs [23], [24]. The early detection and persistence of DTCs in the bone marrow of breast cancer patients signifies the bone marrow microenvironment may function as a reservoir for DTCs [11]. It is highly probable that cancer cells within the bone marrow microenvironment will re-enter the circulation, disseminating to other organs or back to the primary site of tumor formation. Therefore DTCs in the bone marrow not only pose a threat to the development of metastatic lesions in the bone, but may also contribute to the development of metastases at other sites as well as tumor progression and/or recurrence at the primary site.

Although implicated in recurrence at the primary site of tumor formation and the development of metastatic disease, the malignant potential of dormant breast cancer cells residing in the bone marrow remains undetermined. We previously reported detection of early disseminated human breast cancer cells by measuring human DNA in the bone marrow of mice that harbored mammary fat pad tumors derived from injection with primary tumorspheres isolated from patient core biopsies. These early disseminated breast cancer cells were detected prior to the development of metastatic lesions that were detected by H+E staining for up to 12 months post-injections [25]. These findings prompted further investigation into the tumorigenicity and metastatic potential of DTCs within the bone marrow. Herein, we demonstrate a malignant and aggressive metastatic phenotype of dormant breast cancer cells isolated from the bone marrow of mice. These data offer compelling evidence that supports the crucial role of the bone marrow microenvironment in both the maintenance of dormancy and the conversion of breast cancer cells to a more aggressive and rapid growth phenotype once cells have exited the bone microenvironment.

## Materials and Methods

### Cell Culture

Tumorspheres were isolated using a procedure previously described by this laboratory [26] and derived from Dontu et al. [27]. Briefly, breast cancer needle biopsies from primary tumors were obtained during the routine care of patients with consent and Tulane IRB approved protocol (IRB # 07-00042). Tissues were mechanically and enzymatically dissociated then sequentially filtered through a 100 µm and 40 µm pore filter (Fisher). After washes with 1XPBS, the cell pellet was resuspended in DMEM/F12 media containing 1× B-27 serum-free supplement (Invitrogen), 0.4% bovine serum albumin (BSA) (Sigma), 20 ng/ml epidermal growth factor (EGF) (Sigma), 10 ng/ml basic fibroblast growth factor (bFGF) (Sigma), 4 ug/ml insulin, human recombinant (Sigma), and penicillin (100 U/ml)/streptomycin (100 U/ml) and cultured in a 100 mm^2^ ultra low attachment plate (Corning). Cells were cultured for 10–14 days to allow tumorsphere formation. Cells were pelleted every 3 days by centrifugation at 300×g for 10 min. and resuspended in complete DMEM/F12 media supplemented with fresh EGF and bFGF.

### Animal experiments

Immunodeficient Nu/Nu female mice were purchased from Charles River Laboratories (US). Mice were 25–35 days of age at time of injection. All experiments were performed under approved Tulane IACUC protocol (IACUC # 2941 R-D). Cold 1XPBS and a 27 gauge syringe was used to flush bone marrow from the femurs of mice previously injected with tumorspheres isolated from samples 5–9 into the mammary fat pad. Bone marrow was flushed from non-injected age-matched mice and injected into the mammary fat pad as a negative control. The flushed bone marrow was washed twice in cold 1XPBS. To perform a cell count, an aliquot of the isolated bone marrow was combined in a 1∶1 ratio with 0.4% trypan blue stain (BioWhittaker) and loaded onto a hemocytometer. Immediately before injection, the bone marrow was combined with 100 µl BD Matrigel Basement Membrane matrix (BD Biosciences). Mice were anesthetized by i.p. injection of 0.3 ml of a ketamine solution. Cell suspensions were injected bilaterally into the third mammary fat pad. Mice were monitored weekly for tumor formation by caliper measurement and for body weight for up to twelve months. If no weight loss or other indications of declining health were observed, animals were euthanized twelve months post injection.

### Hematoxylin and Eosin (H+E) Staining

Tissues were collected and placed in 10% neutral buffered formalin (Fisher) equal to 20 times the tissue volume. Tissues were incubated overnight at room temperature and then processed by standard formalin fixation, paraffin embedding and sectioning by The Center for Gene Therapy Histology Core Facility, Tulane University Health Sciences Center. 5 µm sections were deparaffinized and rehydrated in a graded series of ethanol solutions, from 100% to 75%. Sections were then stained using Gill's Hematoxylin and Eosin (Poly Scientific) followed by dehydration through a graded series of ethanol solutions from 75% to 100%. Image J software was used to quantify the metastatic burden within the tissues analyzed. To calculate the metastatic burden present in the mouse organs, the number of pixels within the defined area of the metastatic lesion/s was determined (x pixels). Next, the total number of pixels within the field of view was determined (y pixels). The metastatic burden within the field of view was then calculated by dividing the pixels present in the metastatic lesion/s by the total pixels comprising the field of view then multiplying by 100 [(x pixels/y pixels)*100] resulting in a percent metastatic burden. The average of five fields of view (100× magnification) was used to determine metastatic burden present in each organ analyzed.

### Immunohistochemistry (IHC) of tumors and mouse tissue

For IHC, tissues were collected and placed in 10% neutral buffered formalin equal to 20 times the tissue volume (Fisher). Tissues were incubated overnight at room temperature and then processed by standard formalin fixation, paraffin embedding and sectioning by The Center for Gene Therapy Histology Core Facility. IHC was performed using the Vectastain staining kit (anti-rabbit, PK6101; anti-mouse PK6102, Vector Laboratories). Briefly, 5 µm sections were rehydrated (as described above) followed by heat-induced, epitope retrieval performed in a pressure cooker for 25 min. in Tris Buffer, pH 9 (Biocare Medical). To inactivate endogenous peroxide, slides were incubated in 0.3% hydrogen peroxide followed by a 10 minute wash in dH_2_O then washed 3×3 min. each in PBS. Sections were incubated in blocking buffer (10% normal goat serum diluted in PBS) for 30 min. at room temp and subsequently incubated overnight at 4°C with primary antibody diluted in blocking buffer. Primary monoclonal antibodies used were E-cadherin (24E10, Cell Signaling), estrogen receptor α (SP1, Thermo Scientific), and anti-human Human Nuclear Antigen (HNA) (MAB1281, Chemicon). Primary polyclonal antibodies used were β-catenin (9563, Cell Signaling) and fibronectin (ab2413, Abcam). The HNA antibody was human specific. Antibodies for E-cadherin, estrogen receptor α, β-catenin and fibronectin were cross-reactive with human and mouse proteins. The following day, sections were washed 2×5 min. in PBS-T. Biotinylated secondary antibody was added to the sections for an incubation period of 30 min, followed by 2×5 min. washes in PBS-T. Streptavidin/biotin HRP-conjugate was added to the sections for an incubation period of 30 min. at room temperature followed by 2×5 min. washes in PBS-T. The signal was developed for a time that did not exceed 2 minutes using the Vector DAB substrate kit, according to the manufacturers' instructions. Sections were dehydrated (as described above) and mounted using Permount (Fisher). Staining was visualized using a bright field microscope and IP lab software.

## Results

### Tumorigenicity of disseminated cancer cells in the bone marrow of mice previously injected with tumorspheres into the mammary fat pad

Our previous study used tumorspheres derived directly from breast cancer patient needle biopsies to establish primary tumors in the mammary fat pad of nude mice [28]. Two months post-injection of tumorspheres, human cancer cells were detected in the bone marrow of mice without the development of detectable metastatic lesions by H+E staining up to 12 months post-injection [25], suggesting the disseminated breast cancer cells persisted in a dormant state in the bone marrow microenvironment. However, the viability and tumorigenicity of the disseminated breast cancer cells detected in the bone marrow remained undetermined. Therefore, the purpose of this study was to determine the malignant and metastatic potential of the disseminated cancer cells within the bone marrow upon injection into the mammary fat pad.

**Figure 1:**
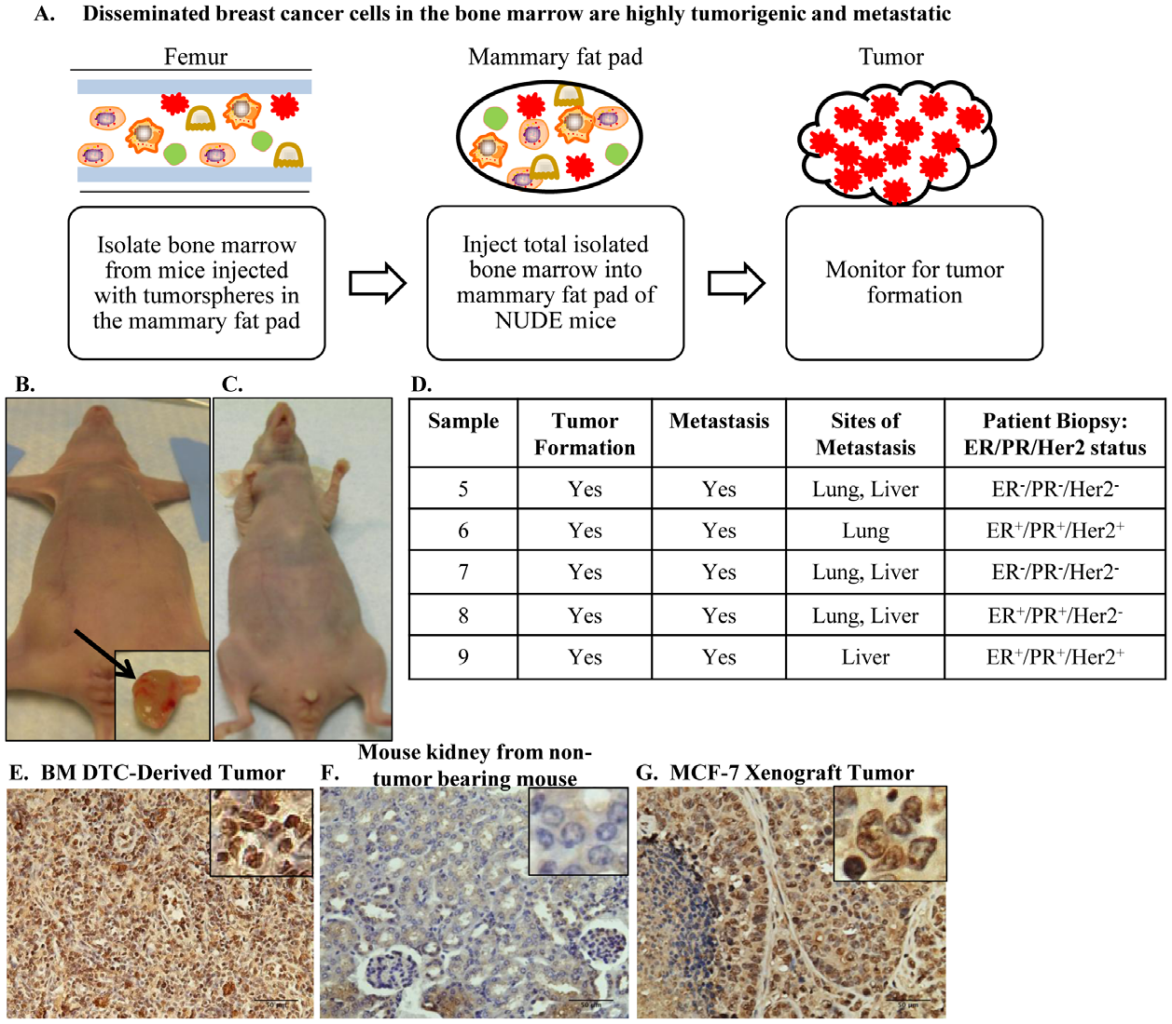
Tumor formation in the mammary fat pad upon injection of total bone marrow aspirates isolated from femurs containing metastatic tumor cells. **A.** Experimental design to determine the tumorigenicity of disseminated human cancer cells in the bone marrow of mice previously injected with tumorspheres into the mammary fat pad. Bone marrow, aspirated from femurs of mice previously injected with tumorspheres into the mammary fat pad, was injected into the mammary fat pad of NUDE mice to determine the tumor-forming ability of dormant cancer cells in the bone marrow. **B.** Injection of 12.5×10^6^ cells/pad aspirated from the femurs of mice injected with tumorspheres resulted in large tumor formation in the mammary fat pad two months post-injection. **C.** Injection of 12.5×10^6^ cells/pad aspirated from the femurs of non-injected mice resulted in no tumor formation in the mammary fat pad three months post injection. **D.** Summary of tumor formation and metastasis for sample 5–9 BM, including ER/PR/Her2 status of patients samples from which the parental tumorspheres were first derived. **E.** Representative positive HNA staining of 5 µm paraffin-embedded sections of sample 5 BM tumors demonstrated the presence of human cells. **F.** No positive nuclear HNA staining of 5 µm paraffin-embedded sections of mouse kidney from non-tumor bearing mouse (negative control). **G.** Positive HNA staining of 5 µm paraffin-embedded sections of a human MCF-7 xenograft (positive control). 200× magnification in all panels.

Tumorspheres isolated from patient biopsy samples 5–9 formed small, palpable tumors upon injection of ≤5×10^3^ cells into the mammary fat pad. These tumors metastasized to the bone (femur), as detected by PCR for human-specific chromosome 17, however did not result in the development of macrometastatic lesions up to 12 months post-injection [25]. Total bone marrow was flushed from the femurs of mice that were injected with tumorspheres isolated from patient core biopsy sample 5–9 into the mammary fat pad 8–10 months prior. 12.5×10^6^ total bone marrow cells (containing normal mouse bone marrow cells and human metastatic breast cancer cells) were injected into the mammary fat pad of NUDE mice (Figure 1A). Injection of 12.5×10^6^ total bone marrow cells from these femurs into the mammary fat pads of mice [referred to as sample 5, 6, 7, 8 or 9 BM (Bone Marrow)] resulted in the formation of large tumors in the mammary fat pad 2 months post-injection (Figure 1B, D). Injection of 12.5×10^6^ total bone marrow cells isolated from non-tumor bearing mice did not result in tumor formation in the mammary fat pad (Figure 1C). To determine whether normal mouse bone marrow cells affected tumor formation by tumorspheres, 12.5×10^6^ bone marrow cells from non-tumor bearing mice were co-injected with sample 5–9 tumorspheres that were previously shown to form small, palpable tumors [25]. Co-injection of normal mouse bone marrow cells with sample 5–9 tumorspheres did not affect primary tumor size (data not shown). Positive staining for HNA on 5 µm paraffin-embedded sections of BM-derived tumors (representative micrograph of positive HNA staining of sample 5 BM tumor, Figure 1E) demonstrated that the majority of cells within the tumors were of human origin. Control sections of 5 µm paraffin-embedded mouse kidney from a non-tumor bearing mouse did not exhibit positive HNA staining (Figure 1F), whereas 5 µm paraffin-embedded sections of a human MCF-7 xenograft tumor exhibited positive HNA staining (Figure 1G) demonstrating the human specificity of the HNA antibody. In addition to being highly tumorigenic, samples 5–9 BM also exhibited metastatic potential with metastatic lesions detected in the lung and liver for sample 5, 7, and 8 BM, metastatic lesions detected in the lung only for sample 6 BM, and metastatic lesions detected in the liver only for sample 9 BM (Figure 1D). These data demonstrate that dormant metastatic breast cancer cells in the bone marrow were highly tumorigenic upon transplantation into the mammary fat pad, forming tumors that were significantly larger than tumors formed by injection of tumorspheres isolated from the original patient biopsies.

**Figure 2:**
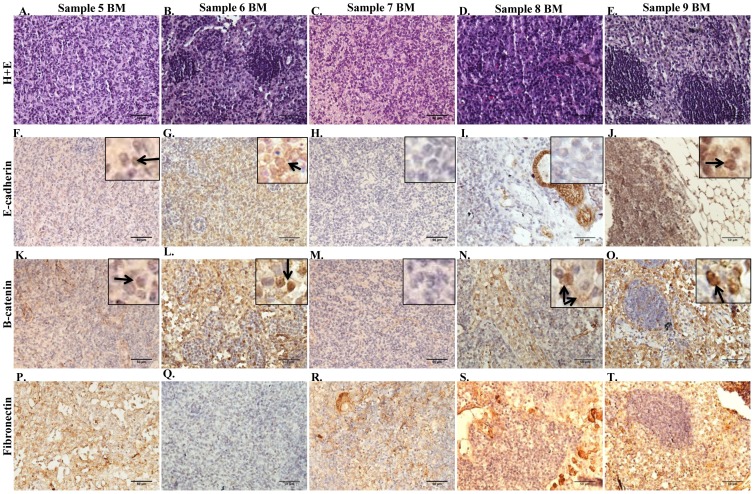
Expression of markers for epithelial and mesenchymal lineages in tumor samples. **A–E.** H+E staining of tumors formed in the mammary fat pad upon injection of bone marrow aspirated from the femurs of mice injected with tumorspheres isolated from samples 5–9 BM, respectively. **F–T.** Representative IHC demonstrating patterns of expression of E-cadherin (**F–J**), β-catenin (**K–O**), and fibronectin (**P–T**) in sample 5–9 BM tumors. 200× magnification in all panels.

The tumors formed from the DTCs present in the injected bone marrow consisted of small tumor cells with pleomorphic nuclei that did not exhibit tubule formation (Figure 2A, B, C, D, E). Using IHC, various markers were evaluated to demonstrate the relative epithelial and mesenchymal features of the tumors. The cell adherens junction protein E-cadherin is normally expressed in the membrane of differentiated epithelial cells and more differentiated breast cancer cells. β-catenin, a central mediator of the WNT signaling pathway, binds to E-cadherin at the membrane in conjunction with a complex of proteins connecting the adherens junction to components of the cytoskeleton [29], [30]. In the absence of membrane E-cadherin, β-catenin is either rapidly degraded or can translocate to the nucleus upon activation of WNT signaling. A low level of E-cadherin expression was variably detected in the nucleus in sample 5, 6 and 9 BM tumors (Figure 2F,G,and J inset, arrow), and no E-cadherin expression was detected in sample 7 and 8 BM tumors (Figure 2H and I). β-catenin expression was variably detected in the membrane and nucleus of sample 5,6, 8 and 9 BM tumors (Figure 2K, L, N, O inset, arrow), with no expression detected in sample 7 BM tumors (Figure 2M). Fibronectin expression was detected in sample 5–9 BM (Figure 2P, Q, R, S, T). Sample 5–9 BM were negative for estrogen receptor alpha (ERα) (data not shown). These data demonstrate that the tumors derived from dormant metastatic breast cancer cells in the bone marrow exhibited a mesenchymal-like phenotype when transplanted into the mammary fat pad of NUDE mice.

### Metastatic potential of disseminated cancer cells in the bone marrow upon injection into the mammary fat pad

**Figure 3:**
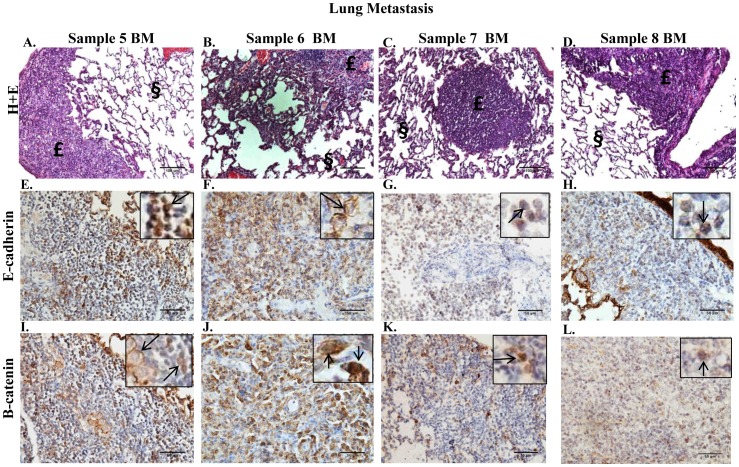
Metastatic lesions in the lungs of mice bearing mammary fat pad tumors that were derived from transplantation of bone marrow aspirate containing metastatic tumor cells. **A–D.** H+E staining performed on 5 µm paraffin-embedded sections of lung from sample 5–8 BM illustrates metastatic lesions. Metastatic lesions indicated by £; normal mouse tissue indicated by §. 100× magnification. **E–H.** IHC performed on 5 µm paraffin-embedded sections of lung from sample 5–8 BM using a monoclonal antibody to E-cadherin. **I–L.** IHC performed on 5 µm paraffin-embedded sections of lung from sample 5–8 BM using a polyclonal antibody to β-catenin.

**Figure 4:**
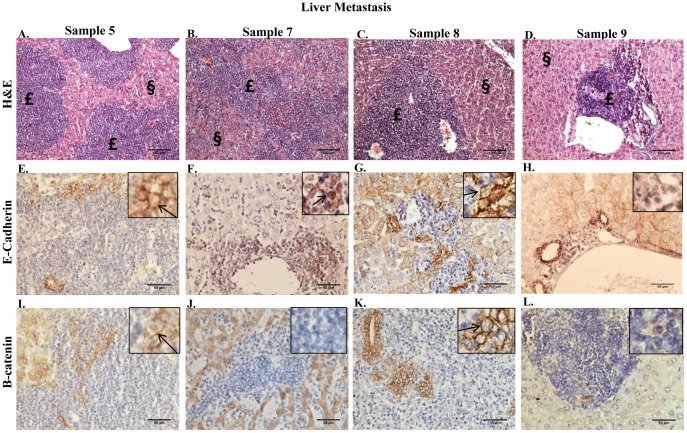
Metastatic lesions in the livers of mice bearing mammary fat pad tumors that were derived from transplantation of bone marrow aspirate containing metastatic tumor cells. **A–D.** H+E staining performed on 5 µm paraffin-embedded sections of liver from sample 5–7, and 9 BM illustrates metastatic lesions. Lesion indicated by £; normal tissue indicated by §. 100× magnification. **E–H.** IHC performed on 5 µm paraffin-embedded sections of liver from sample 5–7, and 9 BM using a monoclonal antibody to E-cadherin. **I–L.** IHC performed on 5 µm paraffin-embedded sections of liver from sample 5–7, and 9 BM using a polyclonal antibody to β-catenin.

Paraffin-embedded sections of lungs, kidneys, and livers were prepared from animals bearing primary tumors from sample 5–9 BM to determine the metastatic potential of the cells. Animals were euthanized for the collection of organs on the basis of tumor burden present in the mammary fat pad and/or declining health. Metastatic lesions were detected by H+E in the lung for sample 5–8 BM (Figure 3A, B, C, D, respectively), in the liver for sample 5, 7–9 BM (Figure 4A, B, C, D, respectively), and in the kidney for sample 5–9 BM (data not shown). Nuclear E-cadherin was detected in the metastatic lesions of the lungs of sample 5, 7 and 8 BM (Figure 3E, G, H, respectively), however E-cadherin was detected predominantly in the membrane in metastatic lesions in the lung of sample 6 BM (Figure 3F). Metastatic lesions in the lung for sample 5 and 6 BM exhibited variable expression of β-catenin in the membrane and in the nucleus (Figure 3I and J, respectively), whereas metastatic lesions in the lung for sample 7 and 8 BM demonstrated variable nuclear expression only of β-catenin (Figure 3K and L, respectively). E-cadherin was detected in the membrane of metastatic cells in the liver of sample 5 and 8 BM (4E and G, respectively); in contrast no E-cadherin expression was detected in liver metastatic lesions of sample 9 BM (Figure 4H). The majority of metastatic cells in the liver for sample 7 BM demonstrated nuclear E-cadherin expression (Figure 4F), however E-cadherin was detected in the membrane of a small population of metastatic cells as well (data not shown). In conjunction with the detection of E-cadherin in the membrane, metastatic cells in the liver for sample 5 and 8 BM exhibited variable β-catenin expression in the membrane (Figure 4I and K, respectively). However, β-catenin expression was not detected in the metastatic cells in the liver for sample 7 and 9 BM (Figure 4J and L, respectively). Fibronectin expression was detected in metastatic lesions in the lung for sample 5–7, and 9 BM and metastatic lesions in the liver for sample 5, 7–9 BM (data not shown). These data indicate that the metastatic cells within the liver for sample 5 and 8 BM exhibited an epithelial-like phenotype, however metastatic cells for sample 5, 7 and 8 BM in the lung and sample 7 and 9 BM in the liver maintained a mesenchymal-like phenotype similarly to that observed within the primary tumors in the mammary fat pad.

### Organ tropism of the metastatic cells and the metastatic burden within the mouse organs

**Figure 5:**
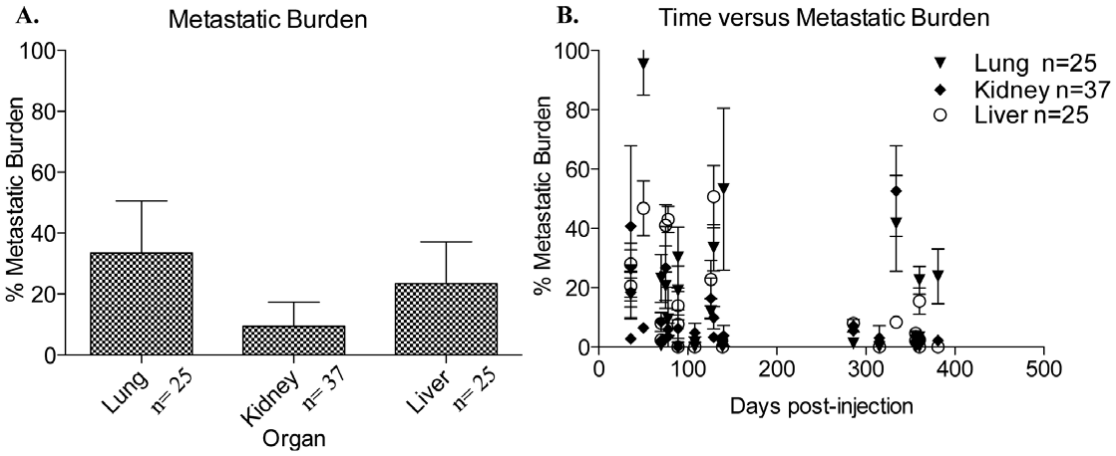
Metastatic profile of bone marrow transplantation experiments. **A.** Graphical representation of the metastatic burden determined for the lungs, kidneys, and livers analyzed by H+E staining from samples 5–9 BM. The metastatic burden in each organ was calculated by dividing the pixels present in the metastatic lesion/s (x pixels) by the total pixels comprising the field of view (y pixels) then multiplying by 100 [(x pixels/y pixels)*100] resulting in a percent value. Values are reported as mean +/− SD. **B.** Graphical representation of the percent metastatic burden, previously calculated as above, compared to days post-injection of total bone marrow into the mammary fat pad for sample 5–9 BM. Values are reported as mean +/− SD.

A comprehensive analysis of metastasis was performed at the time of necropsy (upon excessive tumor burden and/or moribund condition) to compare tropism of each sample to different organs and quantify the relative metastatic burden within each organ for each sample as a measure of the ability of metastatic cells to colonize organ sites with outgrowth into larger lesions. It is likely that metastatic lesions were present prior to termination of the experiments. Future experiments will remove organs at various time intervals post-injection to determine the time to development of metastatic lesions in various organs. To determine differences in tissue-specific tropism between samples, the number of each organ with detectable metastases by H+E staining (lung, kidney and liver) was counted without regard to size of the metastatic lesion. The metastatic burden within each organ was then quantified as described in the material and methods. The number (n) of lungs, kidneys and livers analyzed for each sample is indicated in Figure 5.

The tissue-specific tropism (without regard to the size of the metastatic lesions) was comparable between sample 5–9 BM (data not shown). Sample 5–9 BM exhibited greater metastatic burden within the lung and liver as compared to the kidney, with the overall largest metastatic burden detected in the lung (Figure 5A). In comparison to their parental cell population (tumorspheres isolated from patient core biopsies) for which the average metastatic burden measured for sample 5–9 was below 20%, the DTC-derived metastatic lesions in the lung and the liver exhibited a higher metastatic burden with an average metastatic burden of 35% in the lung and 25% in the liver. The majority of metastatic lesions were detected between 50–150 days post-injection of bone marrow into the mammary fat pad for sample 5–9 BM (Figure 5B). In comparison to the metastatic phenotype demonstrated by sample 5–9 tumors derived from tumorspheres isolated from patient biopsies [25], sample 5–9 BM had a similar overall metastatic phenotype however exhibited an accelerated development of large metastatic lesions.

## Discussion

The bone microenvironment has long been considered to play an important role in the dormancy of disseminated breast cancer cells [11], [15], [31], [32]. However, low numbers of disseminated cells and inaccessibility has hindered studies aimed at elucidating the cellular and molecular mechanisms contributing to dormancy of cancer cells residing in the bone marrow microenvironment. Presented in this study is the first evidence of the malignant potential of dormant breast cancer cells in the bone marrow that had metastasized from a primary tumor in the mammary fat pad derived from primary bTICs. The absence or low expression of epithelial markers, including E-cadherin and β-catenin, and the elevated expression of fibronectin within the tumors indicated that the cells adopted a more mesenchymal phenotype. However, changes in the expression patterns of E-cadherin and β-catenin within metastatic lesions present in the lung and liver suggested that the cells retained a level of plasticity, enabling adaptation and survival at the distant sites of metastasis. The injection of dormant disseminated breast cancer cells present in the bone marrow resulted in the formation of larger primary tumors in the mammary fat pad, and accelerated development (up to 300 days earlier) of large metastatic lesions within the lung, liver and kidney as compared to their “parental” tumorsphere cell population that were derived directly from patient biopsies [25]. Taken together, these data demonstrate that dormant human breast cancer cells residing in the bone marrow microenvironment exhibit a highly malignant and aggressive metastatic phenotype when removed from the bone and transplanted into the mammary fat pad of NUDE mice.

In our previous study, we demonstrated the formation of small tumors in the mammary fat pad upon the injection of tumorspheres isolated directly from patient core biopsies [25]. The tumors became palpable 3 months post-injection and maintained a volume of about 100 mm^3^ for up to 12 months post-injection. From these small tumors, cells disseminated to the bone (femur) and entered a state of dormancy for up to 12 months post-injection, with a small number of cells detected by PCR for human chromosome 17, but without the development of larger metastatic lesions that could be detected by H+E staining. Taking into consideration the malignant capacity of the “parental” population of cells within the tumorspheres, and the dormant state of cells within the bone marrow, it is remarkable that injection of bone marrow containing a small population of disseminated cancer cells into the mammary fat pad resulted in the formation of large tumors within 2 months post-injection. The aggressive malignant phenotype exhibited by dormant tumor cells in bone marrow highlights the importance of the biological changes occurring during metastatic latency, indicating that dormancy is not static but rather the cells are continuously responding and adapting to signals within the microenvironment. Comparing the gene expression profiles of the tumorsphere-derived tumors and BM DTC-derived tumors could reveal the molecular mechanisms contributing to the aggressive phenotype of DTCs post-metastatic latency in the bone marrow. Furthermore, these data indicated a vital role of stromal and/or cellular components within the bone marrow microenvironment for the persistence of disseminated breast cancer cells in a dormant state during metastatic latency.

To determine whether the non-tumorigenic mouse bone marrow was conferring a growth advantage for tumor formation, tumorspheres isolated from sample 5–9 were co-injected into the mammary fat pad with 12.5×10^6^ bone marrow cells isolated from non-injected mice. The co-injection with non-tumorigenic mouse bone marrow did not confer a growth advantage for tumor formation by the tumorspheres; the resulting tumors were similar in size to the small tumors formed by injection of tumorspheres alone. Therefore, the presence of resident mouse bone marrow cells did not account for the malignant phenotype exhibited by the small population of cancer cells present in the isolated bone marrow. However, since the cellular and molecular interactions within the bone marrow microenvironment likely have reciprocal effects on the resident bone marrow cells and the cancer cells, the bone marrow from a non-injected mouse does not control for possible changes in the bone marrow cells.

Previous studies have shown that the majority of DTCs in bone reside within the endosteal niche and vascular niche [33]–[35]. The endosteal and vascular niches are dynamic specialized compartments with cellular and stromal compartments that contribute to the maintenance and differentiation of hematopoietic stem cells (HSCs) [36]–[38]. HSCs are in close association with osteoblasts in the endosteum that together provide signals to maintain HSCs in a primitive, quiescent state and provide an anchor to the endosteal niche [39]–[41]. Migration into the vascular niche from the endosteal niche stimulates the regulated proliferation, differentiation and mobilization of HSCs/HPCs (hematopoietic progenitor cells) to the peripheral circulation [42], [43]. The molecular interactions between the cellular and stromal compartments in the maintenance of HSC quiescence may similarly contribute to the dormancy of cancer cells residing within the endosteal niche. It has been hypothesized that breast cancer dormancy in bone is due to interactions with resident cells within the bone marrow microenvironment, such as mesenchymal stem cells, stromal cells, and osteoblasts (reviewed in [44]). Reactivation of disseminated breast cancer cells for recurrence may occur due to changes in cell-cell signaling, increasing genetic instability, and/or migration of the cancer cells to a different niche within the bone marrow. However, the mechanisms for breast cancer dormancy in bone and tumor recurrence remain unknown. The inherent requirement for the components of the endosteal niche and vascular niche to be responsive to exogenous changes in the environment renders these niches as possible targets for therapeutic manipulation [37]. Investigation into the reciprocal biological changes within the endosteal niche and the cancer cells may elucidate molecular targets within the microenvironment for the eradication of DTCs prior to the development of macroscopic lesions.

The hormone receptor status of the DTCs in the bone marrow is often altered when compared to the original primary tumor. Fehm *et. al.* reported 71% of patients with estrogen receptor alpha (ERα^+^) primary tumors had ERα^−^ DTCs in the BM [45] and Dietsch *et. al.* reported only 2 out of 11 patients with ERα^+^ primary tumors had ERα^+^ DTCs in the BM [46]. In contrast, patients with ERα^−^ primary tumors presented predominantly with ERα^−^ DTCs in the BM [45]. Isolation of tumorspheres from patient samples under non-adherent, serum-free conditions enriches for breast stem/progenitor cells, or breast-tumor-initiating cell (bTICs). It is hypothesized that recurrent and metastatic disease are predominantly derived from the less differentiated cells with stem-like characteristics that would retain the ability to produce progeny with changes in the expression of key markers, resulting in the observed alterations in ERα expression in recurrent and metastatic disease as compared to the primary tumor. Interestingly, both the tumorsphere-derived tumors and their metastases and the DTC-derived tumors and their metastases were negative for ERα despite the expression of ERα in patient tumor samples 6, 8 and 9. Future experiments will determine the expression of ERα within the tumorspheres isolated from the patient samples, as well as the expression of ERα, EMT markers and stem cells markers within early disseminated tumor cells within the bone marrow and other sites of metastasis. Insight into the phenotype of early disseminated tumor cells in the bone marrow could lead to the identification of novel targets for the eradication of DTCs during dormancy prior to the development of recurrent and metastatic disease.

The loss of E-cadherin and β-catenin expression in the membrane of epithelial cells can be indicative of an epithelial to mesenchymal transition (EMT) [47], [48]. It is hypothesized that the metastatic process is initiated when tumor cells undergo either a full or partial EMT, leading to the acquisition of mesenchymal characteristics such as invasiveness, anchorage-independent growth, and resistance to apoptosis [49], [50]. The aberrant and/or low levels of expression of E-cadherin and β-catenin in sample 5–9 BM tumors suggested that the cells with malignant potential acquired some features of a mesenchymal phenotype. If dormant tumor cells residing in the bone marrow microenvironment exhibited a more mesenchymal phenotype, this could have important implications on current techniques employed to detect DTCs in the bone marrow of patients, which predominantly detect epithelial markers such as cytokeratins [7], [17], Her2/neu [51], Epcam [52], and Mucin [6]. The identification of new markers for the detection of biologically relevant DTCs with tumorigenic potential may improve the prognostic value of DTCs in the bone marrow of breast cancer patients in terms of local and distant recurrence.

Metastasis to the lung, liver and kidney demonstrated that the dormant tumor cells within the bone marrow that formed primary tumors in the mammary fat pad retained a similar metastatic profile as the “parental” cells within the tumorspheres; however the expression patterns of E-cadherin and β-catenin within the metastatic lesions in the lung and liver derived from DTC-derived tumors differed from the lesions in the lung and liver derived from tumorsphere-derived tumors [25]. Nuclear localization of E-cadherin has been predominantly observed in pituitary adenomas [53], esophageal squamous cell carcinoma [54], Merkel cell carcinoma [55], solid pseudopapillary tumor of the pancreas [56], [57], clear-cell renal cell carcinoma [58], colorectal cancer (and its liver metastases) [54] and ovarian granulosa cell tumors [59]. Although nuclear localization of E-cadherin has not conclusively been described in breast cancer, one study identified nuclear E-cadherin expression in 21% of FNAC smears from breast carcinomas [60]. Nuclear expression of E-cadherin was found to strongly correlate with higher grade tumors with more aggressive biological behavior in the same study [60]. The down-regulation of E-cadherin in breast tumors is usually a consequence of transcriptional regulation or promoter methylation and is associated with invasion, metastasis and an overall worse prognosis for patients [61]–[64]. However the extracellular and cytoplasmic domain of E-cadherin has been shown to undergo proteolytic cleavage, suggesting another regulatory mechanism of E-cadherin in tumors. Cleavage of the extracellular domain of E-cadherin results in the release of a soluble 80 kDa fragment that has been shown to disrupt cell-cell junctions by antagonizing the full length E-cadherin [65], [66]. In addition, the extracellular fragment of E-cadherin has been shown to bind to and activate Her2 and Her3 cell signaling [67]. Interestingly, cleavage products of E-cadherin were detected by Western blot in protein extracts prepared from DTC-derived tumor samples (Figure S1). Cleavage of E-cadherin at the membrane resulted in an 80 kDa extracellular fragment and 37 kDa intracellular fragment supporting the observation of nuclear localization of E-cadherin by IHC.

MMP-3 and MMP-7 are two proteases implicated in the extracellular cleavage of E-cadherin. Interestingly, the secretion of active MMP-3 and MMP-7 by tumorspheres isolated from samples 5–9 was detected by zymography (unpublished results) however the role of MMP-3 and/or MMP-7 in the aberrant expression of E-cadherin observed in this model has yet to be determined. The cytoplasmic domain of E-cadherin can undergo proteolytic cleavage by caspase-3 and calpain, translocate to the nucleus and influence cell signaling [65], [68]–[70]. However, the mechanisms by which E-cadherin translocates to the nucleus and its potential role in the regulation of gene expression remain unknown. The aberrant expression of E-cadherin detected within the tumorsphere-derived tumors [25], as well as within the DTC-derived tumors and their metastatic lesions in the lung and liver (as presented in this study), provides initial evidence of nuclear E-cadherin in primary breast cancer cells associated with metastasis and other cellular processes. The nuclear localization of E-cadherin warrants further investigation to determine its possible role in tumor progression, metastasis and dormancy. Future studies will be aimed at determining the mechanism by which the cytoplasmic fragment of E-cadherin enters the nucleus, possible interactions with other proteins in the nucleus and any effects on transcription.

The disseminated cancer cells within the bone marrow metastasized to the lung, liver and kidney, as well as the brain and spleen (data not shown). Although the metastatic profiles of the present BM DTC experiments and tumorsphere experiments from our previous study [25] were comparable, there were a few noted differences. The most notable difference between the BM DTC experiments and the tumorsphere experiments [25] was the accelerated development of detectable macrometastatic lesions in the lung, liver and kidney from the BM DTCs. Whereas the majority of metastatic lesions for sample 5–9 were detected at ≥200 days post-injection of tumorspheres into the mammary fat pad [25], the majority of metastatic lesions for sample 5–9 BM were detected at ≤150 days post-injection. The larger primary tumor size and the significantly earlier development of metastases for the BM DTC-derived tumors suggests that the disseminated breast cancer cells in the bone marrow acquired a proliferative advantage through residence in the bone marrow as compared to cells in the tumorsphere-derived tumors. These data may suggest that cells within the bone marrow have the potential to further disseminate to other organs with the proliferative capacity to aggressively form metastatic lesions. Alternatively, the resident bone marrow cells may undergo pre-conditioning as a result of crosstalk with the DTCs or alterations in the microenvironment caused by the presence of the DTCs in the bone. An eloquent study by Kaplan *et. al.* demonstrated the recruitment of VEGFR1^+^ bone marrow derived cells (BMDCs) to tumor-specific sites of metastasis, resulting in the establishment of a pre-metastatic niche for the colonization of disseminated tumor cells [71]. This previous study demonstrated the crucial role of BMDCs in the early steps of metastasis. Therefore, changes within the cells in the bone marrow during metastatic latency may contribute to the highly aggressive metastatic phenotype observed by DTCs in the bone marrow upon injection of total bone marrow into the mammary fat pad. Investigation into the differences between disseminated tumor cells from tumorsphere-derived tumors and BM DTC-derived tumors, as well as changes within the resident bone marrow cells during metastatic latency, may elucidate important pathways involved in cancer cell dormancy.

The organ microenvironment has been implicated in this study as well as previous studies as a possible target in the treatment of metastatic disease. There would be two predominant desired outcomes upon the manipulation of the organ microenvironment: maintenance of dormancy or induced exit from dormancy. On the one hand, the organ microenvironment could be manipulated by exogenous signals to prevent the exit of DTCs from dormancy in an attempt to prevent the development of macroscopic lesions. Alternatively, suppression of the mechanisms contributing to dormancy or activating the signals permitting the exit from dormancy would eliminate metastatic latency allowing the use of cytostatic/cytotoxic therapies to target the proliferating metastatic tumor cells. The results presented in this study suggest that DTCs acquire a highly aggressive and malignant phenotype during metastatic latency indicating that it may be dangerous to permit the persistence of DTCs in a dormant state for extended periods of time. Although cytotoxic and/or cytostatic therapies have been shown to be ineffective in treating metastatic disease, the lack of suitable models has hindered investigation into the efficacy of either approach. Using the model presented in the present study, future research can investigate the treatment of metastatic disease in a systems biology approach, providing a means to determine potential effects on DTCs through manipulation of the organ microenvironment.

Disseminated breast cancer cells in the bone are known to exist in a dormant state for extended periods of time, maintaining the ability to proliferate upon activation to form overt clinical lesions [44], [72], [73]. However the mechanisms contributing to the maintenance of dormancy and subsequent exit from dormancy have yet to be determined. Although the presence of DTCs in the bone marrow provides a strong prognostic indicator for breast cancer, many patients remain relapse-free even after 10 years. In this study, we have presented data implicating the vital role of extrinsic factors within the bone microenvironment in the dormancy of disseminated cancer cells. We demonstrate the malignant potential and aggressive metastatic profile of dormant cancer cells in the bone marrow, suggesting stable modifications of DTCs occur within the bone marrow microenvironment that facilitate malignancy once cells exit the bone. Future studies investigating the reciprocal cross-talk between disseminated cancer cells and resident cells within the bone marrow microenvironment will begin to expose the mechanisms involved in the regulation of cancer cell dormancy that may lead to improved detection and eradication of DTCs in the bone.

## Supporting Information

Figure S1 **Western blot analysis of tumors demonstrating extracellular and intracellular cleavage products of E-cadherin.** Western blot analysis of protein isolated from sample 5 BM tumors demonstrates the presence of the 80 kDa extracellular cleavage product and 37 kDa cytoplasmic cleavage product of E-cadherin.
(TIF)

## References

[1] Husemann Y, Geigl JB, Schubert F, Musiani P, Meyer M, et al (2008) Systemic spread is an early step in breast cancer. Cancer Cell 13: 58–68. 18167340 10.1016/j.ccr.2007.12.003

[2] Engel J, Eckel R, Kerr J, Schmidt M, Furstenberger G, et al (2003) The process of metastasisation for breast cancer. Eur J Cancer 39: 1794–1806. 12888376 10.1016/s0959-8049(03)00422-2

[3] van 't Veer LJ, Dai H, Van De Vijver MJ, He YD, Hart AA, et al (2002) Gene expression profiling predicts clinical outcome of breast cancer. Nature 415: 530–536. 11823860 10.1038/415530a

[4] Schmidt-Kittler O, Ragg T, Daskalakis A, Granzow M, Ahr A, et al (2003) From latent disseminated cells to overt metastasis: genetic analysis of systemic breast cancer progression. Proc Natl Acad Sci U S A 100: 7737–7742. 12808139 10.1073/pnas.1331931100 PMC164657

[5] Coleman RE (1997) Skeletal complications of malignancy. Cancer 80: 1588–1594. 9362426 10.1002/(sici)1097-0142(19971015)80:8+<1588::aid-cncr9>3.3.co;2-z

[6] Diel IJ, Kaufmann M, Costa SD, Holle R, von MG, et al (1996) Micrometastatic breast cancer cells in bone marrow at primary surgery: prognostic value in comparison with nodal status. J Natl Cancer Inst 88: 1652–1658. 8931609 10.1093/jnci/88.22.1652

[7] Braun S, Vogl FD, Naume B, Janni W, Osborne MP, et al (2005) A pooled analysis of bone marrow micrometastasis in breast cancer. N Engl J Med 353: 793–802. 16120859 10.1056/NEJMoa050434

[8] Gebauer G, Fehm T, Merkle E, Beck EP, Lang N, et al (2001) Epithelial cells in bone marrow of breast cancer patients at time of primary surgery: clinical outcome during long-term follow-up. J Clin Oncol 19: 3669–3674. 11504748 10.1200/JCO.2001.19.16.3669

[9] Wiedswang G, Borgen E, Karesen R, Naume B (2003) Detection of isolated tumor cells in BM from breast-cancer patients: significance of anterior and posterior iliac crest aspirations and the number of mononuclear cells analyzed. Cytotherapy 5: 40–45. 12745581 10.1080/14653240310000065

[10] Bidard FC, Vincent-Salomon A, Gomme S, Nos C, de RY, et al (2008) Disseminated tumor cells of breast cancer patients: a strong prognostic factor for distant and local relapse. Clin Cancer Res 14: 3306–3311. 18519757 10.1158/1078-0432.CCR-07-4749

[11] Allgayer H, Aguirre-Ghiso JA (2008) The urokinase receptor (u-PAR)–a link between tumor cell dormancy and minimal residual disease in bone marrow? APMIS 116: 602–614. 18834405 10.1111/j.1600-0463.2008.00997.x PMC2821075

[12] Karrison TG, Ferguson DJ, Meier P (1999) Dormancy of mammary carcinoma after mastectomy. J Natl Cancer Inst 91: 80–85. 9890174 10.1093/jnci/91.1.80

[13] Wiedswang G, Borgen E, Karesen R, Qvist H, Janbu J, et al (2004) Isolated tumor cells in bone marrow three years after diagnosis in disease-free breast cancer patients predict unfavorable clinical outcome. Clin Cancer Res 10: 5342–5348. 15328170 10.1158/1078-0432.CCR-04-0245

[14] Pantel K, Brakenhoff RH (2004) Dissecting the metastatic cascade. Nat Rev Cancer 4: 448–456. 15170447 10.1038/nrc1370

[15] Nicola MH, Bizon R, Machado JJ, Sollero T, Rodarte RS, et al (2003) Breast cancer micrometastases: different interactions of carcinoma cells with normal and cancer patients' bone marrow stromata. Clin Exp Metastasis 20: 471–479. 14524537 10.1023/a:1025462417256

[16] Klein CA, Holzel D (2006) Systemic cancer progression and tumor dormancy: mathematical models meet single cell genomics. Cell Cycle 5: 1788–1798. 3097 [pii]. 16929175 10.4161/cc.5.16.3097

[17] Klein CA, Blankenstein TJ, Schmidt-Kittler O, Petronio M, Polzer B, et al (2002) Genetic heterogeneity of single disseminated tumour cells in minimal residual cancer. Lancet 360: 683–689. 12241875 10.1016/S0140-6736(02)09838-0

[18] Schardt JA, Meyer M, Hartmann CH, Schubert F, Schmidt-Kittler O, et al (2005) Genomic analysis of single cytokeratin-positive cells from bone marrow reveals early mutational events in breast cancer. Cancer Cell 8: 227–239. 16169467 10.1016/j.ccr.2005.08.003

[19] Naume B, Zhao X, Synnestvedt M, Borgen E, Russnes HG, et al (2007) Presence of bone marrow micrometastasis is associated with different recurrence risk within molecular subtypes of breast cancer. Mol Oncol 1: 160–171. 19383292 10.1016/j.molonc.2007.03.004 PMC5543886

[20] Habeck M (2000) Bone-marrow analysis predicts breast-cancer recurrence. Mol Med Today 6: 256–257. 10859556 10.1016/s1357-4310(00)01733-0

[21] Rao G, Patel PS, Idler SP, Maloof P, Gascon P, et al (2004) Facilitating role of preprotachykinin-I gene in the integration of breast cancer cells within the stromal compartment of the bone marrow: a model of early cancer progression. Cancer Res 64: 2874–2881. 15087406 10.1158/0008-5472.can-03-3121

[22] Lim PK, Bliss SA, Patel SA, Taborga M, Dave MA, et al (2011) Gap junction-mediated import of microRNA from bone marrow stromal cells can elicit cell cycle quiescence in breast cancer cells. Cancer Res 71: 1550–1560. 21343399 10.1158/0008-5472.CAN-10-2372

[23] Kim MY, Oskarsson T, Acharyya S, Nguyen DX, Zhang XH, et al (2009) Tumor self-seeding by circulating cancer cells. Cell 139: 1315–1326. 20064377 10.1016/j.cell.2009.11.025 PMC2810531

[24] Leung CT, Brugge JS (2009) Tumor self-seeding: bidirectional flow of tumor cells. Cell 139: 1226–1228. 20064369 10.1016/j.cell.2009.12.013

[25] Marsden CG, Wright MJ, Carrier L, Moroz K, Pochampally R, et al (2012) “A novel in vivo model for the study of human breast cancer metastasis using primary breast tumor-initiating cells from patient biopsies”. BMC Cancer 12: 10. 22233382 10.1186/1471-2407-12-10 PMC3277457

[26] Marsden CG, Wright MJ, Pochampally R, Rowan BG (2009) Breast tumor-initiating cells isolated from patient core biopsies for study of hormone action. Methods Mol Biol 590: 363–375. 19763516 10.1007/978-1-60327-378-7_23

[27] Dontu G, Al Hajj M, Abdallah WM, Clarke MF, Wicha MS (2003) Stem cells in normal breast development and breast cancer. Cell Prolif 36 Suppl 1: 59–72. 14521516 10.1046/j.1365-2184.36.s.1.6.x PMC6495427

[28] Anbalagan M, Ali A, Jones RK, Marsden CG, Sheng M, et al (2012) Peptidomimetic Src/pretubulin inhibitor KX-01 alone and in combination with paclitaxel suppresses growth, metastasis in human ER/PR/HER2-negative tumor xenografts. Mol Cancer Ther 1535–7163. 10.1158/1535-7163.MCT-12-0146 PMC3462004 22784709

[29] Schmalhofer O, Brabletz S, Brabletz T (2009) E-cadherin, beta-catenin, and ZEB1 in malignant progression of cancer. Cancer Metastasis Rev 28: 151–166. 19153669 10.1007/s10555-008-9179-y

[30] Hugo H, Ackland ML, Blick T, Lawrence MG, Clements JA, et al (2007) Epithelial–mesenchymal and mesenchymal–epithelial transitions in carcinoma progression. J Cell Physiol 213: 374–383. 17680632 10.1002/jcp.21223

[31] Feuerer M, Rocha M, Bai L, Umansky V, Solomayer EF, et al (2001) Enrichment of memory T cells and other profound immunological changes in the bone marrow from untreated breast cancer patients. Int J Cancer 92: 96–105. 11279612

[32] Siclari VA, Guise TA, Chirgwin JM (2006) Molecular interactions between breast cancer cells and the bone microenvironment drive skeletal metastases. Cancer Metastasis Rev 25: 621–633. 17165131 10.1007/s10555-006-9023-1

[33] Phadke PA, Mercer RR, Harms JF, Jia Y, Frost AR, et al (2006) Kinetics of metastatic breast cancer cell trafficking in bone. Clin Cancer Res 12: 1431–1440. 16533765 10.1158/1078-0432.CCR-05-1806 PMC1523260

[34] Guise TA, Kozlow WM, Heras-Herzig A, Padalecki SS, Yin JJ, et al (2005) Molecular mechanisms of breast cancer metastases to bone. Clin Breast Cancer 5 Suppl: S46–S53. 15807924 10.3816/cbc.2005.s.004

[35] Kozlow W, Guise TA (2005) Breast cancer metastasis to bone: mechanisms of osteolysis and implications for therapy. J Mammary Gland Biol Neoplasia 10: 169–180. 16025223 10.1007/s10911-005-5399-8

[36] Kopp HG, Avecilla ST, Hooper AT, Rafii S (2005) The bone marrow vascular niche: home of HSC differentiation and mobilization. Physiology (Bethesda ) 20: 349–356. 16174874 10.1152/physiol.00025.2005

[37] Scadden DT (2006) The stem-cell niche as an entity of action. Nature 441: 1075–1079. 16810242 10.1038/nature04957

[38] Morrison SJ, Spradling AC (2008) Stem cells and niches: mechanisms that promote stem cell maintenance throughout life. Cell 132: 598–611. 18295578 10.1016/j.cell.2008.01.038 PMC4505728

[39] Arai F, Hirao A, Ohmura M, Sato H, Matsuoka S, et al (2004) Tie2/angiopoietin-1 signaling regulates hematopoietic stem cell quiescence in the bone marrow niche. Cell 118: 149–161. 15260986 10.1016/j.cell.2004.07.004

[40] Calvi LM, Adams GB, Weibrecht KW, Weber JM, Olson DP, et al (2003) Osteoblastic cells regulate the haematopoietic stem cell niche. Nature 425: 841–846. 14574413 10.1038/nature02040

[41] Nilsson SK, Johnston HM, Coverdale JA (2001) Spatial localization of transplanted hemopoietic stem cells: inferences for the localization of stem cell niches. Blood 97: 2293–2299. 11290590 10.1182/blood.v97.8.2293

[42] Jin DK, Shido K, Kopp HG, Petit I, Shmelkov SV, et al (2006) Cytokine-mediated deployment of SDF-1 induces revascularization through recruitment of CXCR4+ hemangiocytes. Nat Med 12: 557–567. 16648859 10.1038/nm1400 PMC2754288

[43] Abkowitz JL, Robinson AE, Kale S, Long MW, Chen J (2003) Mobilization of hematopoietic stem cells during homeostasis and after cytokine exposure. Blood 102: 1249–1253. 12714498 10.1182/blood-2003-01-0318

[44] Bussard KM, Gay CV, Mastro AM (2008) The bone microenvironment in metastasis; what is special about bone? Cancer Metastasis Rev 27: 41–55. 18071636 10.1007/s10555-007-9109-4

[45] Fehm T, Krawczyk N, Solomayer EF, Becker-Pergola G, Durr-Storzer S, et al (2008) ERalpha-status of disseminated tumour cells in bone marrow of primary breast cancer patients. Breast Cancer Res 10: R76. 18793387 10.1186/bcr2143 PMC2614509

[46] Ditsch N, Mayer B, Rolle M, Untch M, Schildberg FW, et al (2003) Estrogen receptor expression profile of disseminated epithelial tumor cells in bone marrow of breast cancer patients. Recent Results Cancer Res 162: 141–147. 12790328 10.1007/978-3-642-59349-9_12

[47] Berx G, Van Roy F (2001) The E-cadherin/catenin complex: an important gatekeeper in breast cancer tumorigenesis and malignant progression. Breast Cancer Res 3: 289–293. 11597316 10.1186/bcr309 PMC138690

[48] Prasad CP, Rath G, Mathur S, Bhatnagar D, Parshad R, et al (2009) Expression analysis of E-cadherin, Slug and GSK3beta in invasive ductal carcinoma of breast. BMC Cancer 9: 325. 19751508 10.1186/1471-2407-9-325 PMC2753637

[49] Kowalski PJ, Rubin MA, Kleer CG (2003) E-cadherin expression in primary carcinomas of the breast and its distant metastases. Breast Cancer Res 5: R217–R222. 14580257 10.1186/bcr651 PMC314411

[50] Polyak K, Weinberg RA (2009) Transitions between epithelial and mesenchymal states: acquisition of malignant and stem cell traits. Nat Rev Cancer 9: 265–273. 19262571 10.1038/nrc2620

[51] Braun S, Schlimok G, Heumos I, Schaller G, Riethdorf L, et al (2001) ErbB2 overexpression on occult metastatic cells in bone marrow predicts poor clinical outcome of stage I-III breast cancer patients. Cancer Res 61: 1890–1895. 11280743

[52] Pachmann K, Clement JH, Schneider CP, Willen B, Camara O, et al (2005) Standardized quantification of circulating peripheral tumor cells from lung and breast cancer. Clin Chem Lab Med 43: 617–627. 16006258 10.1515/CCLM.2005.107

[53] Elston MS, Gill AJ, Conaglen JV, Clarkson A, Cook RJ, et al (2009) Nuclear accumulation of e-cadherin correlates with loss of cytoplasmic membrane staining and invasion in pituitary adenomas. J Clin Endocrinol Metab 94: 1436–1442. 19158195 10.1210/jc.2008-2075

[54] Salahshor S, Naidoo R, Serra S, Shih W, Tsao MS, et al (2008) Frequent accumulation of nuclear E-cadherin and alterations in the Wnt signaling pathway in esophageal squamous cell carcinomas. Mod Pathol 21: 271–281. 18084253 10.1038/modpathol.3800990

[55] Han AC, Soler AP, Tang CK, Knudsen KA, Salazar H (2000) Nuclear localization of E-cadherin expression in Merkel cell carcinoma. Arch Pathol Lab Med 124: 1147–1151. 10923074 10.5858/2000-124-1147-NLOECE

[56] Chetty R, Serra S (2008) Membrane loss and aberrant nuclear localization of E-cadherin are consistent features of solid pseudopapillary tumour of the pancreas. An immunohistochemical study using two antibodies recognizing different domains of the E-cadherin molecule. Histopathology 52: 325–330. 18269583 10.1111/j.1365-2559.2007.02949.x

[57] Chetty R, Serra S, Salahshor S (2008) E-cadherin in solid pseudopapillary tumors of the pancreas. Hum Pathol 39: 1407–1408. 18706351 10.1016/j.humpath.2008.05.015

[58] Gervais ML, Henry PC, Saravanan A, Burry TN, Gallie BL, et al (2007) Nuclear E-cadherin and VHL immunoreactivity are prognostic indicators of clear-cell renal cell carcinoma. Lab Invest 87: 1252–1264. 17906660 10.1038/labinvest.3700684

[59] Ohishi Y, Oda Y, Kurihara S, Kaku T, Kobayashi H, et al (2011) Nuclear localization of E-cadherin but not beta-catenin in human ovarian granulosa cell tumours and normal ovarian follicles and ovarian stroma. Histopathology 58: 423–432. 21299609 10.1111/j.1365-2559.2011.03761.x

[60] Sauer T, Boudjema G, Jebsen PW, Naess O (2001) Immunocytochemical expression of E-cadherin on fine-needle aspirates from breast carcinomas correlate with the cell dissociation pattern seen on smears. Diagn Cytopathol 25: 382–388. 11747235 10.1002/dc.10030

[61] Salahshor S, Haixin L, Huo H, Kristensen VN, Loman N, et al (2001) Low frequency of E-cadherin alterations in familial breast cancer. Breast Cancer Res 3: 199–207. 11305955 10.1186/bcr295 PMC30704

[62] Cardamone MD, Bardella C, Gutierrez A, Di CL, Rosenfeld MG, et al (2009) ERalpha as ligand-independent activator of CDH-1 regulates determination and maintenance of epithelial morphology in breast cancer cells. Proc Natl Acad Sci U S A 106: 7420–7425. 19383788 10.1073/pnas.0903033106 PMC2671327

[63] Lombaerts M, van Wezel T, Philippo K, Dierssen JW, Zimmerman RM, et al (2006) E-cadherin transcriptional downregulation by promoter methylation but not mutation is related to epithelial-to-mesenchymal transition in breast cancer cell lines. Br J Cancer 94: 661–671. 16495925 10.1038/sj.bjc.6602996 PMC2361216

[64] Hajra KM, Chen DY, Fearon ER (2002) The SLUG zinc-finger protein represses E-cadherin in breast cancer. Cancer Res 62: 1613–1618. 11912130

[65] Marambaud P, Shioi J, Serban G, Georgakopoulos A, Sarner S, et al (2002) A presenilin-1/gamma-secretase cleavage releases the E-cadherin intracellular domain and regulates disassembly of adherens junctions. EMBO J 21: 1948–1956. 11953314 10.1093/emboj/21.8.1948 PMC125968

[66] Wheelock MJ, Buck CA, Bechtol KB, Damsky CH (1987) Soluble 80-kd fragment of cell-CAM 120/80 disrupts cell-cell adhesion. J Cell Biochem 34: 187–202. 3611200 10.1002/jcb.240340305

[67] Najy AJ, Day KC, Day ML (2008) The ectodomain shedding of E-cadherin by ADAM15 supports ErbB receptor activation. J Biol Chem 283: 18393–18401. 18434311 10.1074/jbc.M801329200 PMC2440598

[68] Haas IG, Frank M, Veron N, Kemler R (2005) Presenilin-dependent processing and nuclear function of gamma-protocadherins. J Biol Chem 280: 9313–9319. 15611067 10.1074/jbc.M412909200

[69] Marambaud P, Wen PH, Dutt A, Shioi J, Takashima A, et al (2003) A CBP binding transcriptional repressor produced by the PS1/epsilon-cleavage of N-cadherin is inhibited by PS1 FAD mutations. Cell 114: 635–645. 13678586 10.1016/j.cell.2003.08.008

[70] Rios-Doria J, Day KC, Kuefer R, Rashid MG, Chinnaiyan AM, et al (2003) The role of calpain in the proteolytic cleavage of E-cadherin in prostate and mammary epithelial cells. J Biol Chem 278: 1372–1379. 12393869 10.1074/jbc.M208772200

[71] Kaplan RN, Riba RD, Zacharoulis S, Bramley AH, Vincent L, et al (2005) VEGFR1-positive haematopoietic bone marrow progenitors initiate the pre-metastatic niche. Nature 438: 820–827. 16341007 10.1038/nature04186 PMC2945882

[72] Welch DR, Cooper CR, Hurst DR, Lynch CC, Martin MD, et al (2008) Metastasis Research Society-American Association For Cancer Research Joint Conference on Metastasis. Cancer Res 68: 9578–9582. 68/23/9578. 19047132 10.1158/0008-5472.CAN-08-3360 PMC2741417

[73] Barkan D, Kleinman H, Simmons JL, Asmussen H, Kamaraju AK, et al (2008) Inhibition of metastatic outgrowth from single dormant tumor cells by targeting the cytoskeleton. Cancer Res 68: 6241–6250. 18676848 10.1158/0008-5472.CAN-07-6849 PMC2561279

