# Supplementary figures and images for: Disseminated Breast Cancer Cells Acquire a Highly Malignant and Aggressive Metastatic Phenotype during Metastatic Latency in the Bone
Source: PLoS One. 2012 Nov 15;7(11):e47587. doi: 10.1371/journal.pone.0047587 (PMC3500091; doi:10.1371/journal.pone.0047587)

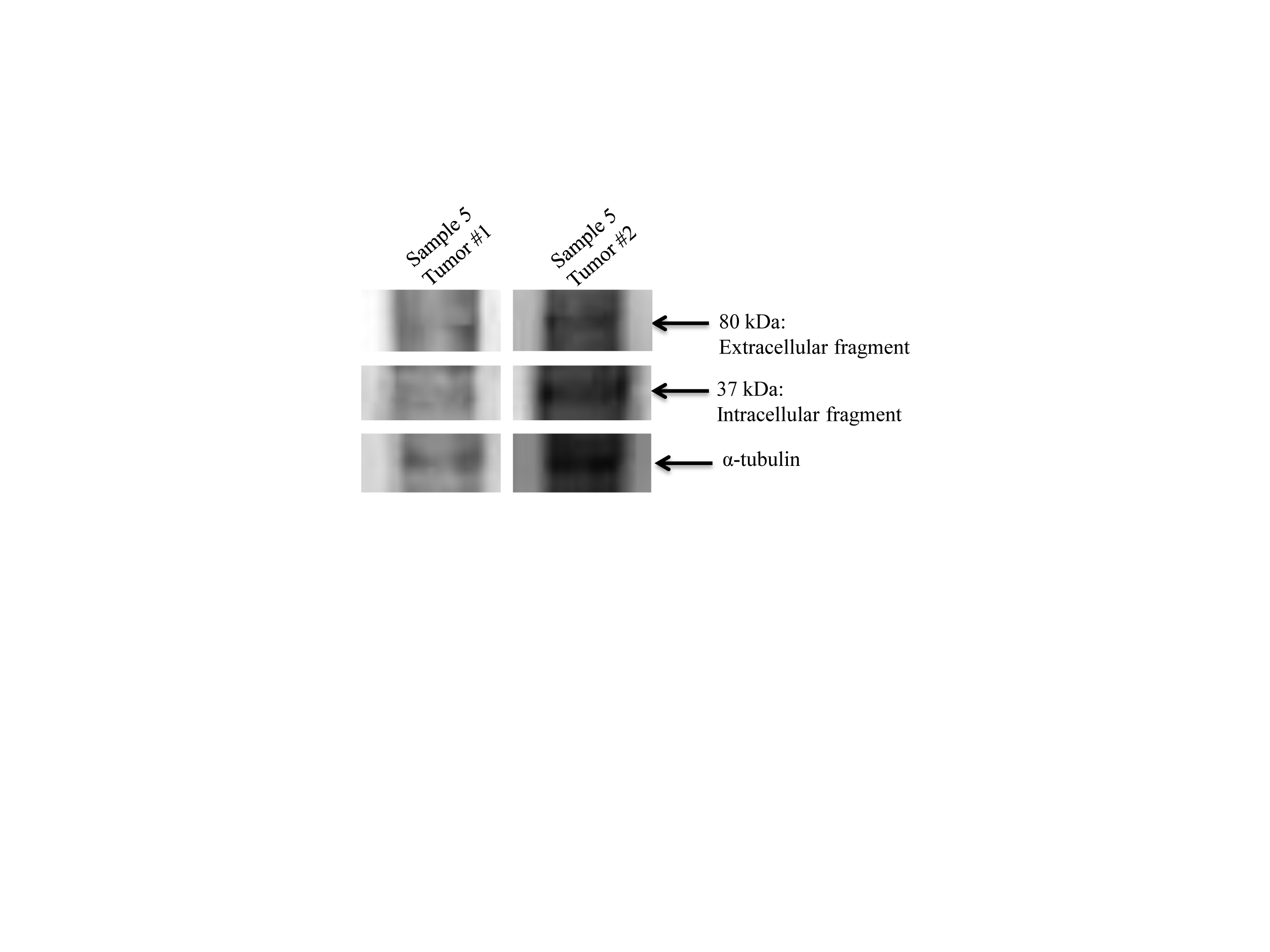

Supplement: Figure S1 — Western blot analysis of tumors demonstrating extracellular and intracellular cleavage products of E-cadherin. Western blot analysis of protein isolated from sample 5 BM tumors demonstrates the presence of the 80 kDa extracellular cleavage product and 37 kDa cytoplasmic cleavage product of E-cadherin. (TIF) [file pone.0047587.s001.tif]
